# Stringent monitoring can decrease mortality of immune checkpoint inhibitor induced cardiotoxicity

**DOI:** 10.3389/fcvm.2024.1408586

**Published:** 2024-06-10

**Authors:** Ying Wang, Carolin Ertl, Christina Schmitt, Linda Hammann, Rafaela Kramer, Ulrich Grabmaier, Florian Schöberl, David Anz, Ignazio Piseddu, Giulia Pesch, Julio Vera, Waltraud Froehlich, Ludwig Weckbach, Dirk Tomsitz, Carmen Loquai, Lisa Zimmer, Johanna Mangana, Reinhard Dummer, Ralf Gutzmer, Kai-Christian Klespe, Henner Stege, Frank Meiss, Kai-Martin Thoms, Patrick Terheyden, Paul J. Bröckelmann, Douglas B. Johnson, Lars E. French, Lucie Heinzerling

**Affiliations:** ^1^ Department of Dermatology and Allergy, University Hospital, LMU Munich, Munich, Germany; ^2^ SERIO Registry, Munich, Germany; ^3^ Division of Clinical Pharmacology, Klinikum der Universität München, Munich, Germany; ^4^ Department of Dermatology, Friedrich-Alexander University Erlangen-Nürnberg (FAU) and University Hospital Erlangen (UKER), Deutsches Zentrum Immuntherapie (DZI) and Comprehensive Cancer Center Erlangen-European Metropolitan Area of Nürnberg (CCC-ER-EMN), Erlangen, Germany; ^5^ Department of Medicine I, LMU University Hospital, LMU Munich, Munich, Germany; ^6^ Department of Neurology, LMU University Hospital, LMU Munich, Munich, Germany; ^7^ German Center for Vertigo and Balance Disorders (DSGZ), LMU University Hospital, LMU Munich, Munich, Germany; ^8^ Department of Medicine II, LMU University Hospital, LMU Munich, Munich, Germany; ^9^ Department of Dermatology, Klinikum Bremen-Ost, Gesundheit Nord gGmbH, Bremen, Germany; ^10^ Department of Dermatology, University Hospital Essen & German Cancer Consortium (DKTK), Partner Site Essen/Duesseldorf, & National Center for Tumor Diseases (NCT)-West, Campus Essen, & Research Alliance Ruhr, Research Center One Health, University Duisburg-Essen, Essen, Germany; ^11^ Department of Dermatology, University Hospital Zurich, Zurich, Switzerland; ^12^ Department of Dermatology, Johannes Wesling Medical Center, Mühlenkreiskliniken (MKK), Ruhr University Bochum, Minden, Germany; ^13^ Skin Cancer Center Hannover, Department of Dermatology and Allergy, Hannover Medical School, Hannover, Germany; ^14^ Department of Dermatology, University Medical Center of the Johannes Gutenberg University Mainz, Mainz, Germany; ^15^ Faculty of Medicine, Department of Dermatology, Medical Center—University of Freiburg, Freiburg, Germany; ^16^ Department of Dermatology, University Medical Center Goettingen, Georg-August-University, Goettingen, Germany; ^17^ Department of Dermatology, University of Lübeck, Lübeck, Germany; ^18^ Department I of Internal Medicine, Center for Integrated Oncology Aachen Bonn Cologne Duesseldorf, University of Cologne, Cologne, Germany; ^19^ Department of Medicine, Vanderbilt University Medical Center, Nashville, TN, United States; ^20^ Dr. Philip Frost, Department of Dermatology and Cutaneous Surgery, University of Miami Miller School of Medicine, Miami, FL, United States

**Keywords:** checkpoint inhibitor, melanoma, immunotherapy, myocarditis, cardiovascular toxicity

## Abstract

**Background:**

Immune checkpoint inhibitor (ICI)-induced myocarditis is a rare immune-related adverse event (irAE) with a fatality rate of 40%–46%. However, irMyocarditis can be asymptomatic. Thus, improved monitoring, detection and therapy are needed. This study aims to generate knowledge on pathogenesis and assess outcomes in cancer centers with intensified patient management.

**Methods:**

Patients with cardiac irAEs from the SERIO registry (www.serio-registry.org) were analyzed for demographics, ICI-related information (type of ICI, therapy line, combination with other drugs, onset of irAE, and tumor response), examination results, irAE treatment and outcome, as well as oncological endpoints. Cardiac biopsies of irMyocarditis cases (*n* = 12) were analyzed by Nanostring and compared to healthy heart muscle (*n* = 5) and longitudinal blood sampling was performed for immunophenotyping of irMyocarditis-patients (*n* = 4 baseline and *n* = 8 during irAE) in comparison to patients without toxicity under ICI-therapy (*n* = 4 baseline and *n* = 7 during ICI-therapy) using flow cytometry.

**Results:**

A total of 51 patients with 53 cardiac irAEs induced by 4 different ICIs (anti-PD1, anti-PD-L1, anti-CTLA4) were included from 12 centers in 3 countries. Altogether, 83.0% of cardiac irAEs were graded as severe or life-threatening, and 11.3% were fatal (6/53). Thus, in centers with established consequent troponin monitoring, work-up upon the rise in troponin and consequent treatment of irMyocarditis with corticosteroids and –if required–second-line therapy mortality rate is much lower than previously reported. The median time to irMyocarditis was 36 days (range 4–1,074 days) after ICI initiation, whereas other cardiotoxicities, e.g. asystolia or myocardiopathy, occurred much later. The cytokine-mediated signaling pathway was differentially regulated in myocardial biopsies as compared to healthy heart based on enrichment Gene Ontology analysis. Additionally, longitudinal peripheral blood mononuclear cell (PBMC) samples from irMyocarditis-patients indicated ICI-driven enhanced CD4+ Treg cells and reduced CD4+ T cells. Immunophenotypes, particularly effector memory T cells of irMyocarditis-patients differed from those of ICI-treated patients without side effects. LAG3 expression on T cells and PD-L1 expression on dendritic cells could serve as predictive indicators for the development of irMyocarditis.

**Conclusion:**

Interestingly, our cohort shows a very low mortality rate of irMyocarditis-patients. Our data indicate so far unknown local and systemic immunological patterns in cardiotoxicity.

## 1 Background

Immune checkpoint inhibitors (ICI) have revolutionized tumor therapy and are effective in multiple tumor entities (1, 2). However, they also induce a broad spectrum of immune-related toxicities, including colitis, hepatitis, pneumonitis, thyroiditis, myositis, hypophysitis, dermatitis, and cardiotoxicity (3–6). ICI-related myocarditis (irMyocarditis) emerged as a significant concern, occurring in 0.5%–1% of ICI recipients (7–10), with a median onset of 34 days post-treatment initiation (11). Alarmingly, irMyocarditis historically carried a mortality rate of 40%–46%, which is the highest of any irAE and 10-fold higher than myocarditis from other causes (12, 13). The clinical presentation of irMyocarditis ranges from asymptomatic troponin elevation to heart failure, with ventricular arrhythmia, severe conduction disorders, or cardiogenic shock and may co-occur with symptoms of myositis and myasthenia gravis (3, 14).

Even though, early, and high dose steroids seem to be associated with a lower risk of death, the outcome of cardiac irAE is often hard to predict (15, 16). To ensure diagnosis, current guidelines recommend conducting various examinations and, in case of cardiovascular complications, the referral to cardiology (6, 17, 18). Up to now, diagnostics, monitoring, and therapy for cardiac irAEs under immunotherapy are still largely based on experience or, if one exists, guided by the clinic's internal algorithm. Therefore, monitoring for cardiac events comes with a large burden of unnecessary assessments and a reliable evidence base for treatment options is still lacking. IrMyocarditis can be induced by activation of autoreactive T-lymphocytes directed against heart muscle (19). The target antigens may overlap with striated muscle and thus create an overlap of irMyocarditis, irMyositis and myasthenia-like syndrome (14). Recently, single-cell RNA sequencing coupled with T cell receptor analysis revealed the enrichment of cytotoxic T cells, inflammatory macrophages, and conventional dendritic cells in heart tissue biopsies from patients with irMyocarditis (13). Notably, cardiac and tumor infiltrates from melanoma patients with fatal irMyocarditis shared high-frequency T cell receptor sequences, suggesting potential tumor-expressed antigen triggers for ICI-induced myocarditis (20). Autopsy specimens from patients with irMyocarditis have demonstrated cardiac infiltration primarily by T-lymphocytes and macrophages, with notable absence of B cells or antibody deposits (20). Another study identified an increase of CD8+ effector memory T-cells re-expressing CD45RA (T_EMRA_) in peripheral blood of irMyocarditis patients based on time-of-flight mass cytometry (CyTOF) (21). This would suggest that targeting cytotoxic CD8+ T cells may be effective in treating fulminant myocarditis.

Tissue transcriptomics of irMyocarditis has rarely been reported to date. In one study, bulk RNA sequencing of myocardial tissue from patients with irMyocarditis indicated that irMyocarditis was associated with multiple inflammatory pathways, especially interferon responses. Several interferon-stimulated genes were upregulated, including CXCL9, MDK, and GBP521 (22). In a murine study, CD8+ T cells of mice with myocarditis demonstrated a unique transcriptional profile consisting of proinflammatory and cytotoxicity markers (GZMB, GNLY, CST7, NKG7, KLRB1, and IL32), and myocardial-tropic chemokines CCL5, CCL4, and CCL4L2 (21).

This study analyzed a multicenter cohort of patients with cardiac irAEs with respect to symptoms, outcome and longitudinal changes of immunophenotype in comparison to patients without irAEs. Additionally, gene expression analyses of the corresponding heart muscle biopsies were conducted.

## 2 Method and design

### 2.1 Study population and design

This retrospective multicenter study includes a total of 51 patients from 12 centers (Cologne, Erlangen, Essen, Freiburg, Goettingen, Hannover, Luebeck, Mainz, Minden, Munich, Nashville, Zurich) with confirmed cardiac irAE drawn from our SERIO-registry and was approved by the ethics committee of the LMU University Hospital Munich (No. 20-1122). The diagnosis of irMyocarditis was confirmed by cardiologists at the corresponding center, who assessed symptoms, ECG alterations, troponin levels, and functional or structural changes in cardiac imaging, alongside a confirmed temporal association with the administration of ICIs. Cardiac MRIs were conducted on 33 patients, revealing findings consistent with irMyocarditis, such as late gadolinium enhancement, myocardial edema, wall motility disorders, and/or ischemia. Furthermore, endomyocardial biopsy results from 18 patients showed evidence of toxic damage to myocardial cells, including interstitial fibrosis, degenerative changes, and increased infiltration of lymphocytes, especially T cells, and CD68-positive macrophages, further supporting the diagnosis. If available, we collected creatine kinase (CK), creatine phosphokinase-MB (CK-MB), troponin T, and N-terminal prohormone of brain natriuretic peptide (NT-proBNP) serum levels at peak, as well as examination results of electrocardiogram (ECG), echocardiography (Echo) with left ventricular ejection fraction (LVEF), cardiac magnetic resonance imaging (MRI), and coronary angiography.

Clinical data was gathered using the international web-based Side Effect Registry Immuno-Oncology (SERIO; www.serio-registry.org). It was initiated in cooperation with the Paul-Ehrlich-Institute, to document rare, complex, or therapy-refractory side effects induced by immunotherapies. Within SERIO patients’ demographics, tumor entity, ICI therapy applied, tumor outcome, as well as type of irAE, irAE onset, grade, treatment and outcome are documented. The grading of irAEs followed the Common Terminology Criteria for Adverse Events (CTCAE) version 5.0. IrAE outcome was classified as resolved, improved, resolved with sequelae, or ongoing. The classification of completely “resolved” cardiac irAEs refers to cases where all cardiac symptoms and abnormalities associated with the irAE have disappeared. “Improved” defines irAE cases where there is a noticeable enhancement in cardiac irAE symptoms and/or abnormalities but which haven't entirely disappeared. “Resolved with sequelae” indicates that while the symptoms have subsided, there are residual effects or complications of the cardiac irAE, e.g., elevated troponin levels, remaining. “Ongoing” signifies cardiac irAE cases where symptoms and abnormalities persist without any significant improvement or resolution. Ethical approval for analyses from the SERIO registry was granted (Erlangen Nr. 2_20 B, Erlangen Nr. 17_16 Bc). SERIO was queried for cases of cardiac irAEs in February 2024.

For lab investigations participants gave their informed written consent prior to analysis. In total, peripheral blood mononuclear cells (PBMCs) from 19 patients were analyzed longitudinally for immunophenotyping at baseline and during ICI-therapy or during irAE. PBMCs from irMyocarditis patients (*n* = 4 baseline and *n* = 8 during irAE) were compared to patients without toxicity under ICI-therapy (*n* = 4 baseline and *n* = 7 during ICI-therapy). Heart muscle biopsies included irMyocarditis patients from Erlangen, Luebeck, Munich (D), and Zuerich (CH) (*n* = 11 available for lab investigations). Healthy heart muscle biopsies were obtained from pediatric cardiac surgery (*n* = 5). The healthy muscle was from a biobank that captured heart muscle from patients with valve reconstruction.

### 2.2 Blood sample processing and flow cytometry

PBMCs were isolated from lithium heparin blood samples by density gradient centrifugation with Ficoll-Paque density-gradient centrifugation followed by a cryopreservation with X-VIVO medium (Lonza) enriched with 20% fetal bovine serum (FBS, Pan Biotech) as well as with 10% DMSO (Thermo Fisher) and stored in liquid nitrogen. PBMCs were obtained from 8 patients with irMyocarditis and 11 ICI-patients without development of toxicities.

Thawed PBMCs were rested for one hour in RPMI medium + 10% FCS + 1% Pen/Strep + 1% L-Glutamine. Prior to staining, cells were incubated with human TrueStain Fc blocking reagent (Biolegend). Cells were then incubated (30 min, 4 °C) with eBioscience Fixable Viability Dye eFluor506 (Invitrogen) and the surface antibody mix (Table 1) in PBS. After fixation and permeabilization with eBioscience fixation/permeabilization reagent (Invitrogen; 30 min, 4 °C) and washing, cells were incubated (30 min, 4 °C) with the intracellular/intranuclear antibody mix (Table 1) and then measured the same day on an LSR Fortessa Cell Analyzer (BD Biosciences). FlowJo software (10.10.1) (BioSciences) was used for analysis.

**Table 1:** The list of FACS antibodies used for PBMC.

Antigen	Fluorophore	Clone	Company
CD25	BV421	BC96	biolegend
CD8	BV510	SK1	biolegend
CD127	BV605	A019D5	biolegend
CD103	BV711	Ber-ACT8	biolegend
CD45RA	FITC	HI100	biolegend
CD4	PerCP-Cy5.5	SK3	biolegend
FOXP3	PE	259D	biolegend
CD62l	PE/Dazzle 94	DREG-56	biolegend
CD45RO	PE-Cy7	UCHL1	biolegend
CCR7	APC	G043H7	biolegend
CD3	AF700	OKT3	biolegend
TIGIT	BV421	A15153G	biolegend
ICOS	BV605	C398.4A	biolegend
TIM-3	BV650	F38-2E2	biolegend
CTLA4	BV711	BNI3	biolegend
CD44	FITC	C44Mab-5	biolegend
CD107a	PE	H4A3	biolegend
LAG-3	PE/Dazzle 594	11C3C65	biolegend
CD28	PE-Cy7	CD28.2	biolegend
CD27	APC	M-T271	biolegend
CD80	BV421	2D10	biolegend
CD16	BV510	B73.1	biolegend
BDCA1	BV605	L161	biolegend
BDCA2	BV711	201A	biolegend
CD11c	FITC	Bu15	biolegend
PD-L1	PerCP-Cy5.5	29E.2A3	biolegend
CD86	PE	BU63	biolegend
CD14	PE/Dazzle 594	HCD14	biolegend
HLA-DR	PE-Cy7	L243	biolegend
CD19	APC	HIB19	biolegend
CD56	AF700	5.1H11	biolegend
Mouse IgG1	BV421	MOPC-21	biolegend
Mouse IgG1	BV510	MOPC-21	biolegend
Mouse IgG1	BV605	MOPC-21	biolegend
Mouse IgG1	BV650	MOPC-21	biolegend
Mouse IgG1	BV711	MOPC-21	biolegend
Mouse IgG2b	FITC	MPC-11	biolegend
Mouse IgG1	PerCP-Cy5.5	MOPC-21	biolegend
Mouse IgG1	PE	MOPC-21	biolegend
Mouse IgG1	PE/Dazzle 94	MOPC-21	biolegend
Mouse IgG2a	PE-Cy7	MOPC-173	biolegend
Mouse IgG2a	APC	MOPC-173	biolegend
Mouse IgG2a	AF700	MOPC-173	biolegend
Mouse IgG2a	BV421	MOPC-173	biolegend
Armenian hamster IgG	BV605	HTK888	biolegend
Mouse IgG2a	BV711	MOPC-21	biolegend
Mouse IgG1	FITC	MOPC-21	biolegend
Mouse IgG2b	PerCP-Cy5.5	MPC-11	biolegend
Mouse IgG1	PE-Cy7	MOPC-21	biolegend
Mouse IgG1	APC	MOPC-21	biolegend
Mouse IgG1	AF700	MOPC-21	biolegend

Low cytometry antibodies used for FACS analysis are listed with respect to antigen, fluorophore, clone and company.

### 2.3 RNA isolation

Total RNA was isolated from heart muscle specimens using TRIzol™ Reagent (Thermo Fisher Scientific, Germany) according to the manufacturer's instructions.

### 2.4 Nanostring analysis

Gene expression was analyzed using the nCounter PanCancer Immune Profiling Panel™ (human) (Nanostring, XT-CSO-HIP1–12). Sample detection and analysis were completed on a nCounter® Digital Analyzer. RNA gene expression analysis was performed using the nCounter SPRINT Profiler and the nCounter MAX Analysis System (NanoString Technologies, Inc.). Statistical analysis of gene expression results was implemented with the nSolver Analysis Software (NanoString Technologies, Inc.) and ROSALIND® (ROSALIND, Inc., San Diego, CA) (https://rosalind.bio/). Pathway analysis was conducted using EnrichR and EnrichR-KG, applying the databases KEGG 2021 Human and GO Biological Process 2021.

### 2.5 Unsupervised and statistical analysis

Raw data processing, quality control and normalization were performed using the NanoStringQCPro package for the R (version 4.1.0) environment. Quality control (QC) was performed with an imaging QC of >80% field of view registration, binding density QC within 0.05–2.25 range, and positive control scaling factors within a range of 0.3–3. In the differential expression analysis, a false discovery rate (FDR, Benjamini and Hochberg) adjusted *p*-value of ≤0.05 was applied as cutoff. One sample two-tailed unpaired Student's *t*-test, one sample Wilcoxon test and Mann–Whiney *U*-test were used to test statistical significance. *: *p* ≤ 0.05, **: *p* ≤ 0.01, and ***: *p* ≤ 0.001.

## 3 Results

**Figure 1:**
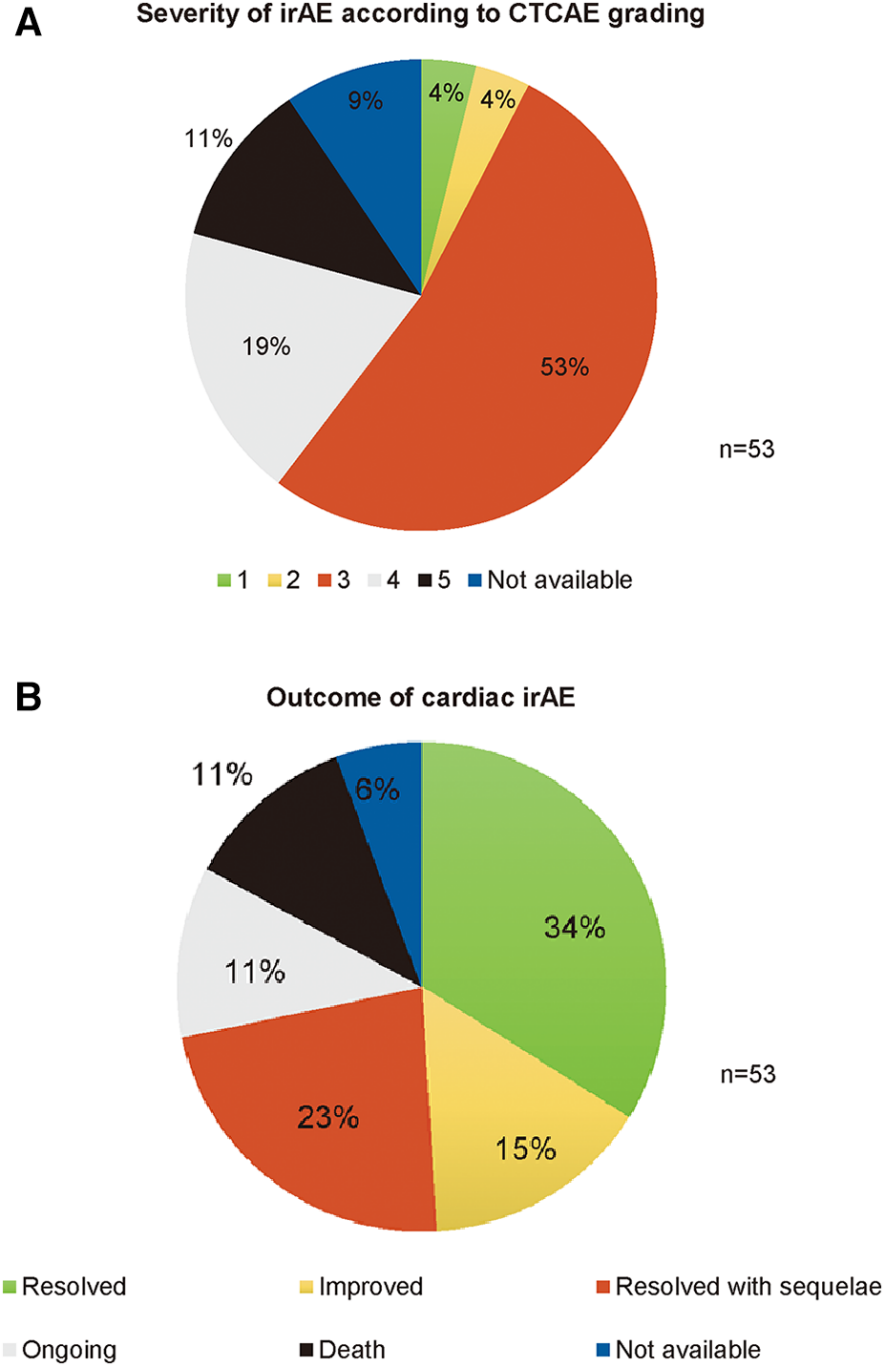
Documented cardiac irAE severity and outcome within the side effect registry immuno-oncology (SERIO). (**A**) Severity of irAE (grade 1-5 CTCAE). Data was available for 90.6% (48/53) of cases. (**B**) Outcome of irAE. Outcome data was available for 94.3% (50/53) of cases. CTCAE, common terminology criteria for adverse events version 5.0; IrAE, immune-related adverse event.

In total, 51 patients (19 female—32 male patients; 37.2%–62.7%) with various tumor entities (Table 2) were included in this study investigating cardiac irAE. Most patients were diagnosed with cutaneous melanoma (31/51; 60.7%), followed by uveal melanoma (4/53; 7.8%) and melanoma of unknown primary (MUP) (3/51; 5.9%). Other reported tumor entities included, but were not limited to, head and neck carcinoma (2/51; 3.9%), neuroendocrine bladder cancer (5/51; 3.9%); breast cancer (1/51; 2.0%) and renal cell carcinoma (1/51; 2.0%). Applied checkpoint inhibitors included anti-cytotoxic T lymphocyte antigen 4 (CTLA-4), anti-programmed cell death 1 (PD-1), and anti-programmed cell death 1 ligand 1 (PD-L1) antibodies. Most frequently applied ICI regimens were combined therapy with ipilimumab and nivolumab (21/53; 39.6%), pembrolizumab (15/53; 28.3%) and nivolumab (11/53; 20.8%) in monotherapy. Reported cardiac irAEs included myocarditis (48/53; 90.5%), myocardiopathy (1/53; 1.9%), myocardial fibrosis (1/53; 1.9%), pericarditis (1/53; 1.9%), ventricular arrhythmia (1/53; 1.9%) and asystolia (1/53; 1.9%). Altogether, 53 cardiac irAE were documented, since 2 of 51 patients developed a second cardiac irAE. Three quarter of patients showed another irAE (72.5%; 37/51) with irMyositis (15/52; 28.8%) and irHepatitis (13/52; 24.5%) being the most frequently reported ones. According to CTCAE grading, 83.0% of cardiac irAEs were severe or life-threating (Grade ≥ 3 adverse events) (Figure 1) and 11.3% were fatal (6/53). IrMyocarditis, in particular, was fatal in 8.3% of cases (4/48). Death due to tumor progression occurred in 31.4% (16/51) of patients upon longitudinal analysis.

**Table 2:** Baseline characteristics of patients with immune-related cardiac adverse events associated with immunotherapy.

Patients’ characteristics	Patients with cardiac irAE
*n* (patients)	51
*n* (cardiac irAE)	53
*n* (all irAE)	105
Age (years), *n*	70 (23–91)
Sex:	19–32
Female—male: *n* (%)	(37%–63%)
Tumor entity	
Melanoma; cutaneous	31 (60.7%)
Melanoma of unknown primary (MUP)	3 (5.9%)
Melanoma; uveal	4 (7.8%)
Melanoma; amelanotic	2 (3.9%)
Squamous cell carcinoma (SCC)	2 (3.9%)
Breast cancer	1 (2.0%)
Head and neck carcinoma	2 (3.9%)
Neuroendocrine bladder cancer	2 (3.9%)
Non-small-cell lung cancer (NSCLC)	1 (2.0%)
Renal cell carcinoma (RCC)	1 (2.0%)
Sigmoid carcinoma	1 (2.0%)
Uterine cancer	1 (2.0%)
Melanoma	
Primary AJCC (2017) stage, *n* (%)	
I	0 (0%)
II	1 (2.6%)
III A	0 (0%)
III B/C	5 (13.2%)
IV	32 (84.2%)
Cardiovascular risk factors, *n* (%)	
Arterial hypertension	17 (38.6%)
High cholesterol	7 (15.9%)
Diabetes mellitus type 2	7 (15.9%)
Smoking	9 (20.5%)
Obesity	4 (9.1%)
Therapy regimen ICI, *n* (%)	
Cemiplimab	2 (3.8%)
Ipilimumab	2 (3.8%)
Ipilimumab + Nivolumab	21 (39.6%)
Ipilimumab + Pembrolizumab	1 (1.9%)
Nivolumab	11 (20.8%)
Pembrolizumab	15 (28.3%)
Pembrolizumab ± TVEC	1 (1.9%)
ICI combination, *n* (%)	
Yes	22 (41.5%)
No	31 (58.5%)
Duration of ICI treatment, *n* (%)	
Continued	1 (1.9%)
Interrupted	12 (22.2%)
Stopped	36 (66.7%)
Rechallenge	5 (9.3%)*
Cardiac irAE, *n* (%)	
Myocarditis	48 (90.6%)
Asystolia	1 (1.9%)
Myocardial fibrosis	1 (1.9%)
Myocardiopathy	1 (1.9%)
Pericarditis	1 (1.9%)
Ventricular arrhythmia	1 (1.9%)
Other irAE, *n* (%)	**37** (**72.5%)**
Myositis	15 (28.8%)
Hepatitis	13 (24.5%)
Thyroiditis	5 (9.6%)
Colitis	4 (7.5%)
Other	3 (5.8%)
Hypophysitis	2 (3.8%)
Nephritis	2 (3.8%)
Pneumonitis	2 (3.8%)
Rheumatological irAE	2 (3.8%)
Auditory/vestibular irAE	1 (1.9%)
Hematological irAE	1 (1.9%)
Lichenoid skin reactions	1 (1.9%)
Ocular irAE	1 (1.9%)
Cardiac irAE, CTCAE grade *n* (%)	
1–2	4 (8.3%)
3–5	44 (91.6%)
Cause of death, *n* (%)	
Tumor progression	16 (72.7%)
Cardiac irAE	6 (27.3%)

Percentages may not sum up to 100 due to rounding. Age: median displayed with range in brackets. Primary AJCC 2017 stage and irAE displayed. IrAE are graded according to Common Terminology Criteria for Adverse Events Version 5.0 of 2017.

CTCAE, common terminology criteria for adverse events version 5.0; ICI, immune-checkpoint-inhibitor; irAE, immune-related adverse event; TVEC, talimogene laherparepvec.

* 4 patients with no recurrence of cardiac irAE; 1 fatality.

### 3.1 Signs and symptoms

The median time from initiation of ICI therapy to onset of cardiac irAEs was 37 days with a wide range (4–1,074 days). The time to onset of irMyocarditis significantly differed from other cardiac irAEs. In total, irMyocarditis occurred early after ICI initiation with a median time to onset of 36 days (range 4–1,074 days), whereas other cardiac irAEs had a median time to onset of 101 days (range 28–119 days). More than half of all patients (58.8%; 30/51) initially presented with symptoms like dyspnea (63.3%; 19/30) or chest pain (23.3%; 7/30) while 41.2% were asymptomatic. In over 50.0% of irAE cases, which ultimately resolved completely, elevated laboratory parameters (Troponin T, CK, CK-MB, and NT-proBNP) were initially observed (Table 3). In those patients that fully recovered, echocardiographic findings were predominantly within the normal range (92.9%; 13/14) and coronary angiographies presented with no signs of stenosis (75.0%; 9/12). The results of ECG and cardiac MRI in this cohort were nonspecific, showing a distribution of 50.0% abnormal and 50.0% normal results. Notably, biopsies of the myocardium of patients who fully recovered were indicative of myocarditis in 7 out of 8 cases (87.5%). Interestingly, in the cohort that ultimately led to fatality, cardiac diagnostic assessments were not performed in more than 50.0% of cases. Upon conducting the investigations in this cohort, abnormalities were observed in ECG (100.0%; 3/3) and coronary angiography (100.0%; 2/2), whereas the echocardiogram (66.7%; 2/3), LVEF (100.0%; 3/3), and MRI (100.0%; 1/1) findings were predominantly within the normal range (Table 3).

**Table 3:** Findings in patients with cardiac irAE.

Findings in patients with cardiac irAE	Outcome of cardiac irAE	
*n* (cardiac irAE)	Resolved (*n* = 18)	Death (*n* = 6)
Symptoms		
Yes	11 (61.1%)	4 (66.7%)
No	5 (27.8%)	0 (0%)
Not performed or n/a	2 (10.5%)	2 (33.3%)
Laboratory results		
Troponin		
Elevated	14 (77.8%)	3 (50.0%)
Normal	0 (0%)	0 (0%)
Not performed or n/a	4 (22.2%)	3 (50.0%)
CK		
Elevated	12 (66.7%)	3 (50.0%)
Normal	4 (22.2%)	0 (0%)
Not performed or n/a	2 (11.1%)	3 (50.0%)
CK-MB		
Elevated	9 (50.0%)	2 (33.3%)
Normal	2 (11.1%)	0 (0%)
Not performed or n/a	7 (38.9%)	4 (66.7%)
NT-proBNP		
Elevated	9 (50.0%)	2 (33.3%)
Normal	3 (16.7%)	0 (0%)
Not performed or n/a	6 (33.3%)	4 (66.7%)
Further examinations		
ECG		
Abnormal	7 (38.9%)	3 (50.0%)
Normal	7 (38.9%)	0 (0%)
Not performed or n/a	4 (22.2%)	3 (50.0%)
Echo		
Abnormal	1 (5.6%)	1 (16.7%)
Normal	13 (72.2%)	2 (33.3%)
Not performed or n/a	4 (22.2%)	3 (50.0%)
LVEF		
Reduced	2 (11.1%)	0 (0%)
Normal	12 (66.7%)	3 (50.0%)
Not performed or n/a	4 (22.2%)	3 (50.0%)
Coronary angiography		
Stenosis	3 (16.7%)	2 (33.3%)
No stenosis	9 (50.0%)	0 (0%)
Not performed or n/a	6 (33.3%)	4 (66.7%)
Cardiac MRI		
Abnormal	7 (38.9%)	0 (0%)
Normal	6 (33.3%)	1 (16.7%)
Not performed or n/a	5 (27.8%)	5 (83.3%)
Biopsy of myocardium		
Yes	7 (38.9%)	0 (0%)
No	1 (5.6%)	0 (0%)
Not performed or n/a	10 (55.6%)	6 (100%)

The results express the analysis of two patient cohorts categorized by cardiac irAE outcomes “Resolved” and “Death” and the standard assessments conducted for cardiac irAE, including the number of cases with abnormal or normal results, as well as the cases that were not investigated.

CK, creatine kinase; CK-MB, creatine phosphokinase-MB; cMRI, cardiac magnetic resonance imaging; ECG, electrocardiogram; Echo, echocardiograpy; irAE, immune-related adverse event; LVEF, left ventricular ejection fraction; NT-proBNP, N-terminal prohormone of brain natriuretic peptide.

### 3.2 IrAE treatment and outcome

In total, 92.5% (49/53) of patients with cardiac irAE were treated with systemic steroids, 5.7% (3/53) were solely treated with symptomatic therapies such as beta blockers and antihypertensive drugs, and 17.0% (9/53) required second-line therapy for steroid-refractory or steroid-dependent cardiac irAEs. As second-line immunosuppressants, intravenous immunoglobulins (IVIG), infliximab, anti-thymocyte globulin (ATG), mycophenolate mofetil (MMF) were applied. Optimal response rates of 90.0% (4/5) were attained through the administration of IVIG. In total, 72.5% (37/51) of patients required hospitalization.

Data evaluation of cardiac irAE outcomes indicated that 34.0% (18/53) of cases completely resolved, 15.1% (8/53) significantly improved, while 22.6% (12/53) resolved with relevant sequelae and 11.3% (6/53) were still ongoing, with an overall irAE response rate of 71.7% (Figure 1). In 11.3% (6/53) of cases, cardiac irAEs led to death (Figure 1). In our study, 5 patients received ICI rechallenge after cardiac irAE. In 80.0% (4/5) of cases ICI was successfully applied without recurrence of cardiac toxicities, while in one case the patient died early after ICI re-initiation due to exacerbated cardiac symptoms. In one case, ICI rechallenge was successfully administered with prophylactic steroid treatment. Tumor response in patients with cutaneous melanoma was assessed at the time of irAE: Complete response was reported in 5.9% (2/34), partial response in 8.8% (3/34), stable disease in 20.6% (7/34) and progressive disease in 32.4% (11/34) of patients (RECIST 1.1). Patients showed a median PFS of 8 months (range 0–60 months) and a median overall survival (OS) of 17 months (range 0–130 months). In total, 11.8% (4/34) of patients were treated in an adjuvant setting with recurrence-free survival between 1 and 13 months.

### 3.3 Gene expression analysis reveals substantial differences in cytokine pathways between irMyocarditis and healthy controls

**Figure 2:**
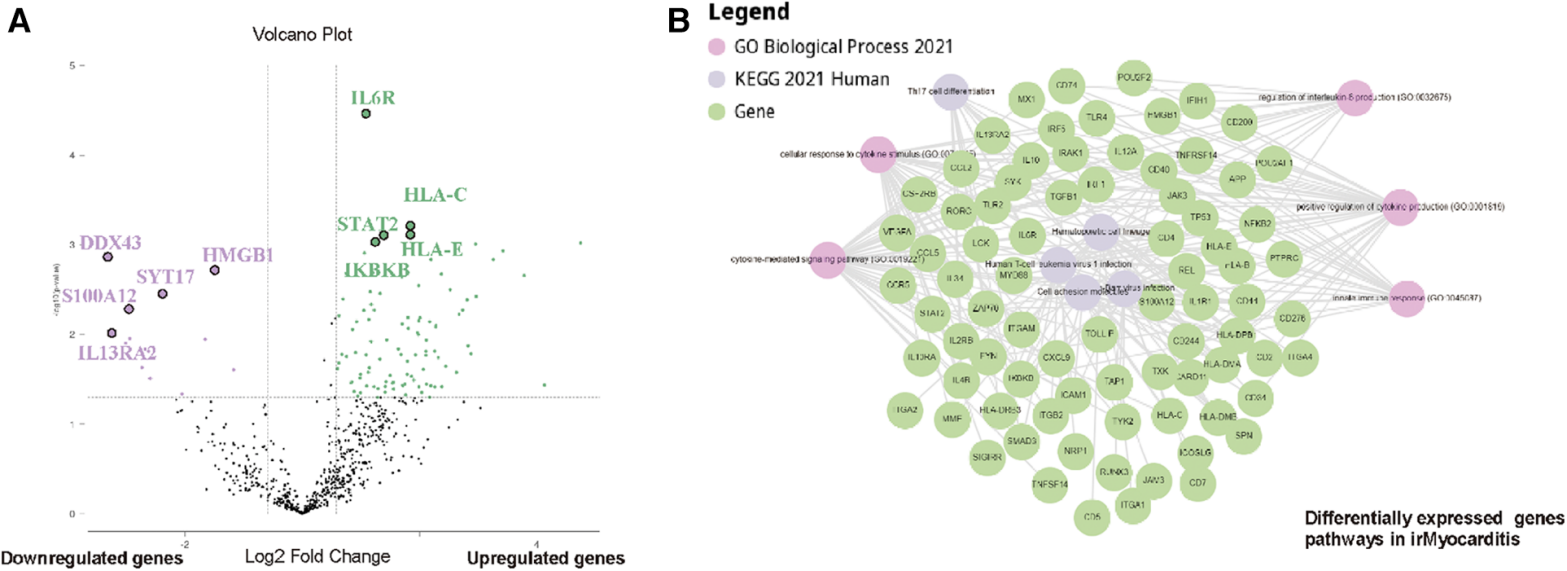
Bulk RNAseq reveals substantial differences in cytokine production pathways between irMyocarditis and healthy control. (**A**) Volcano plot shows 14 genes significantly downregulated in in irMyocarditis cohort and healthy heart cohort (violet) and 99 genes significantly upregulated in irMyocarditis compared to healthy control (green) (Log2 fold change ≤−1.5 or ≥1.5, *p*-value ≤0.05). Statistics were performed using Rosalind® software. (**B**) Enriched pathway analysis of significantly differentially expressed genes performed with STRING®, gene ontology and KEGG® reveals networks of enriched genes in irMyocarditis cohort and healthy heart cohort.

To elucidate the gene expression signatures of irMyocarditis, heart tissue of 12 irMyocarditis patients was compared to heart tissue of 5 healthy controls using nCounter® Digital Analyzer. After normalizing the raw data, with a ratio >1.5 or <1.5 and *p* < 0.05 as the screening criteria for differential expression genes (DEG), the DEGs analysis identified a set of 113 differentially regulated genes in irMyocarditis compared to healthy controls, of which 99 genes were upregulated and 14 were downregulated (Figure 2A). The top five upregulated genes in irMyocarditis were IL6R (logFC 2.1, adjusted *p*-value 3.34 × 10^−5^), HLA-C (logFC 3.62, adjusted *p*-value 6.31 × 10^−4^), HLA-E (logFC 3.58, adjusted *p*-value 7.19 × 10^−4^), STAT2 (logFC 2.64, adjusted *p*-value 8.25 × 10^−4^) and IKBKB (logFC 2.41, adjusted *p*-value 9.22 × 10^−4^). The top five downregulated genes in irMyocarditis were DDX43 (logFC −10.04, adjusted *p*-value 3.01 × 10−4), HMGB1 (logFC −2.75, adjusted *p*-value 1.90 × 10^−3^), SYT17 (logFC −5.17, adjusted *p*-value 3.64 × 10^−3^), S100A12 (logFC −7.79, adjusted *p*-value 5.43 × 10^−3^) and IL13RA2 (logFC −9.45, adjusted *p*-value 9.85 × 10^−3^). The 113 DEGs were investigated using gene set enrichment and pathway analysis (Figure 2B), demonstrating the most significant pathways of the DEGs: (1) Cytokine-mediated signaling pathway (*p* = 6.6445 × 10^−37^), (2) Cellular response to cytokine production (*p* = 5.5129 × 10^−32^), (3) Positive regulation of cytokine production (*p* = 1.4357 × 10^−30^), (4) Hematopoietic cell lineage (*p* = 1.4458 × 10^−22^), (5) Epstein-Barr virus infection (*p* = 2.5618 × 10^−22^), (6) Cell adhesion molecules (*p* = 2.858 × 10^−22^), (7) Th17 cell differentiation (*p* = 2.6598 × 10^−20^), (8) Human T-cell leukemia virus 1 infection (*p* = 3.6892 × 10^−20^), (9) Regulation of interleukin-6 production (*p* = 1.724 × 10^−15^), (10) Innate immune response (*p* = 6.7096 × 10^−15^). Thus, the regulation of cytokines, especially type I IFNs and NFkB signaling was differently shaped in irMyocarditis compared to healthy control, indicating might also be relevant—IL-6 can be therapeutically inhibited.

### 3.4 Enhanced activation of Treg cells and dendritic cells related to irMyocarditis development

**Figure 3:**
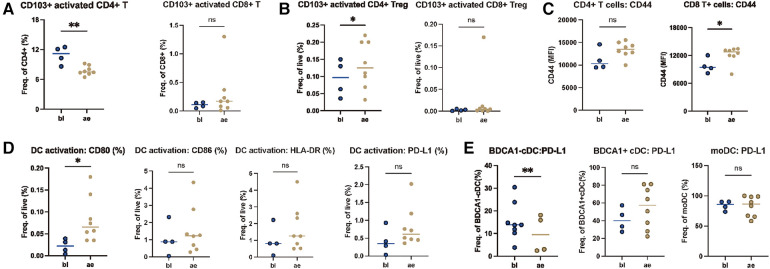
Enhanced activated Treg cells driven by ICI in irMyocarditis patient. PBMCs of patients with ae-irMyocarditis (*n* = 8) and bl (*n* = 4) were analyzed via flow cytometry. Distribution of activated T cells, Treg cells and other T cell phenotypes (Tn = naive T cells, Tcm = central memory T cells, Tem = effector memory T cells, Teff = effector T cells) in CD4+ and CD8+ T cells was determined. CD4+ and CD8+ T cells were also analyzed for expression of activation (CD27, CD28, ICOS, CD107a, CD44) and exhaustion (CTLA4, TIM-3, TIGIT, LAG3) markers using FACS. The abundance and activation of dendritic cell (DC) subsets was analyzed [activated DC, BDCA1- DC, BDCA1+ DC and monocyte derived DC (moDC)] using flow cytometry. (**A**) The frequencies of CD4+ and CD8+ in CD103+ activated CD4+ T cells and CD103+ activated CD8+ T cell. (**B**) The frequencies of live cells in CD103+ activated CD4+ Treg cells and CD103+ activated CD8+ Treg cells. (**C**) CD4+ and CD8+ T cells were analyzed for expression of activation CD44 markers using FACS. (**D**) The frequencies of live cells in CD80+, CD86+, HLA-DR, PD-L1 activated DC cells. (**E**) The frequencies of BDCA1- DC in PD-L1+ BDCA1- DC, PD-L1+ BDCA1+ DC and PD-L1+ moDC. IrAE, immune-related adverse event; bl, baseline.

Based on the analyzed gene expression patterns of immune response mechanisms, we next focused on characterization of the profile of the systemic immune response. We performed longitudinal analysis of PBMCs from irMyocarditis patients (baseline and time of adverse event). In longitudinal analysis, PBMCs of patients during irMyocarditis (ae) demonstrated significantly decreased frequencies of CD103+ activated CD4+ T cells and increased CD103+ activated CD4+ Treg cells compared to baseline (bl) whereas activated T cells as well as Treg cells showed no significant difference between bl and ae. Furthermore, no significant difference in CD103+ activated CD8+ T cells and activated CD8+ Treg cells was observed. Integrin CD103 is related to tissue residency in the context of inflammation and cancer (Figures 3A,B). The peripheral T cell compartment was investigated to determine the relative abundance of various T cell phenotypes, including effector-like T cells (Teff), effector memory-like T cells (Tem), central memory-like T cells (Tcm), and naïve T cells (Tn). This analysis utilized established markers such as CD45RA, CD45RO, CD62l, CCR7, and CD127. The frequency of Tn, Tcm, Tem, and Teff showed no significant difference neither in CD4+ nor in CD8+ T cells (Supplementary Figures S1A,B). Furthermore, markers associated with T cell activation (CD44, CD27, CD28, ICOS, and CD107a) and exhaustion (CTLA4, LAG3, TIM3, and TIGIT) were examined to gain further functional insights into differences in systemic T cell immunity. The CD8+ T cells showed an increased expression of CD44 which was not observed on CD4+ T cells (Figure 3C). Furthermore, we focused on the function and phenotype of antigen-presenting cells (APCs). We determined three different DC subsets in peripheral blood including BDCA1- cDC, BDCA1+ cDC, and monocyte-derived DC (moDC) based on the markers CD11c, CD19, CD56, CD14, BDCA1, and HLA-DR and determined the expression of the activation markers CD80, CD86 and HLA-DR as well as the inhibitory molecule PD-L1. Regarding DC, we found significantly higher frequencies of activated DC during the adverse event compared to baseline in irMyocarditis patient's PBMC as determined by the expression of the costimulatory molecule CD80 (Figure 3D). Besides, for DC subsets, the expression of the coinhibitory molecule PD-L1 on BDCA1- cDC was significantly reduced in ae compared to bl, indicating the effect might be related to the development of irMyocarditis (Figure 3E). The expression of CD80, CD86, and the MHC class II molecule HLA-DR on BDCA1- cDC, BDCA1+ cDC, and moDC showed no significant difference between ae and bl (Supplementary Figures S1C–E). To summarize, the peripheral T cell compartment of irMyocarditis patients showed ICI-driven enhanced levels of activated CD4+ Treg cells, CD44, CD80, PD-L1 expression on DC, and reduced levels of activated CD4+ T cells.

### 3.5 Tem cell and exhaustion markers dominate in irMyocarditis compared to patients without toxicity

**Figure 4:**
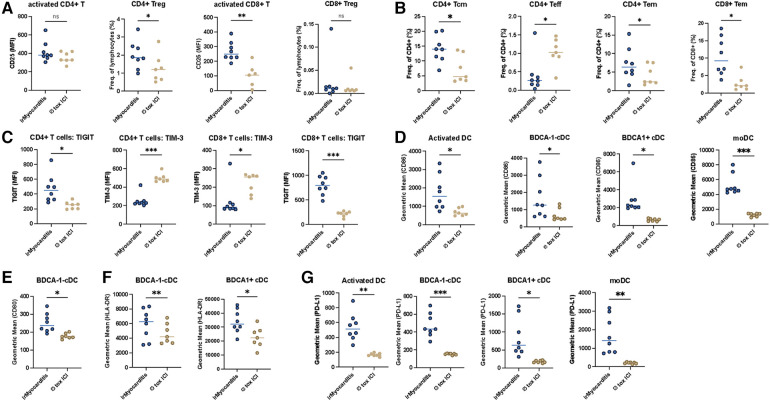
Tem cell and exhaustion markers dominate in the induction of cardiotoxicity in irMyocarditis patients. PBMCs of patients with irMyocarditis (*n* = 8) and patients undergoing checkpoint-inhibitor therapy without development of toxicities (Ø tox ICI, *n* = 7) were analyzed via flow cytometry. Distribution of activated T cells, Treg cells, Tn, Tcm, Tem, Teff in CD4+ and CD8+ T cells was determined. CD4+ and CD8+ T cells were also analyzed for expression of activation (CD27, CD28, ICOS, CD107a, CD44) and exhaustion (CTLA4, TIM-3, TIGIT, LAG3) markers using FACS. The abundance and activation of dendritic cell (DC) subsets were analyzed using FACS. (**A**) The expression level of CD25 in activated CD4+, CD8+ T cell and the frequencies of lymphocytes of CD4+ Treg, CD8+ Treg cells (**B**) The frequencies of CD4 in CD4+ Tcm, CD4+ Teff, CD4+ Tem, and CD8+ Tem. (**C**) The expression level of TIGIT, TIM3 in CD4+ and CD8+ T cells. (**D**) The expression level of CD86 in activated DC, BDCA1- DC, BDCA1+ DC cells. (**E**) The expression level of CD80 of BDCA1- DC. (**F**) The expression level of HLA-DR of BDCA1- DC, BDCA1+ DC. (**G**) The frequencies of PD-L1 in activated DC, BDCA1- DC, BDCA1+ DC and moDC.

Following the longitudinal immunophenotype changes in irMyocarditis patients from baseline to adverse event, we analyzed the irMyocarditis patients during the adverse events in comparison to patients who did not develop toxicities while undergoing ICI-therapy toxicities (Ø tox ICI-patients). PBMCs of irMyocarditis patients showed significantly higher frequencies of CD4+ Treg cells and higher levels of activated CD8+ T cells compared to Ø tox ICI-patients (Figure 4A). Interestingly, different from previous results, irMyocarditis patients showed significantly higher frequencies of CD4+ Tcm cells and CD4+ Teff cells compared to Ø tox ICI-patients. Furthermore, we observed a significant decrease in CD4+ Tem cells and CD8+ Tem cells in irMyocarditis compared to Ø tox ICI-patients (Figure 4B), whereas no significant alterations of CD4+ naïve T cells and CD8+ naïve T cells could be observed (Supplementary Figure S2A). Noticeably, among the tested functional markers only CD28 of CD4+ T cells was elevated in Ø tox ICI-patients (Supplementary Figures S2B,C). Of note, both CD4+ as well as CD8+ T cells of irMyocarditis patients showed significantly increased expression of the T cell exhaustion marker TIGIT, however a decrease of TIM-3 expression on CD8+ T cells (Figure 4C). For other T cell exhaustion markers, the expression of LAG3 was elevated in CD4+ T cells but not in CD8+ T cells in irMyocarditis compared to Ø tox ICI-patients (Supplementary Figure S2D). The expression of CTLA4, however, was not different in irMyocarditis and Ø tox ICI-patients neither in CD4+ nor in CD8+ T cells (Supplementary Figure S2E). Further analysis of APC cells showed significantly higher expression of CD86 on activated DC, BDCA1- cDC, BDCA1+ cDC, and moDC (Figure 4D). Regarding other costimulatory molecules, we found significantly higher frequencies of CD80 on BDCA1- DC as well as the HLA-DR on BDCA1- cDC, BDCA1+ cDC in irMyocarditis patients, pointing towards an increase in cDC activation potentially induced by irMyocarditis (Figures 4E,F). The expression of CD80 markers on activated DC, BDCA1+ DC, and moDC, and the expression of HLA-DR on moDC and activated DC, however, was not statistically different between irMyocarditis and Ø tox ICI-patients (Supplementary Figures S2F,G). Strikingly, the expression of PD-L1 showed a significant increase in all DC subsets in irMyocarditis patients (Figure 4G). Furthermore, in our irMyocarditis cohort, circulating B cells were significantly less abundant than in Ø tox ICI-patients (Supplementary Figure S2H). Lastly, a difference between CD56high/CD16- NK cells and CD56low/CD16+ NK cells could not be detected between irMyocarditis and Ø tox ICI-patients, which indicated the cardiotoxicity might not be related to NK cell-mediated cytotoxicity (Supplementary Figure S2I). Taken together, these data suggest that irMyocarditis is characterized by activation of T cells, particularly Tem cells, additionally the exhaustion markers TIGIT, TIM and LAG3 were identified to be of greater importance in irMyocarditis patients.

### 3.6 LAG3 and PD-L1 as potential indicators to predict the occurrence of irMyocarditis

**Figure 5:**
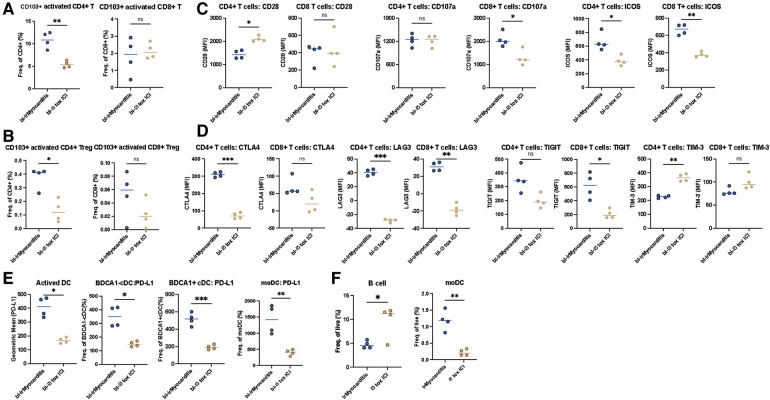
LAG3 and PD-L1 can be used as indicators to predict the occurrence of irMyocarditis. PBMCs of patients with bl-irMyocarditis (*n* = 4) and bl-tox ICI (*n* = 4) were analyzed via flow cytometry. Distribution of activated T cells, Treg cells, Tn, Tcm, Tem, Teff in CD4+ and CD8+ T cells was determined. CD4+ and CD8+ T cells were also analyzed for expression of activation (CD27, CD28, ICOS, CD107a, CD44) and exhaustion (CTLA4, TIM-3, TIGIT, LAG3) markers using FACS. The abundance and activation of dendritic cell (DC) subsets were analyzed using FACS. (**A**) The frequencies of CD4, CD8 in activated CD4+, CD8+ T cells. (**B**) The frequencies of CD4, CD8 in CD103+ activated CD4+ Treg, CD8+ Treg cells (**C**) The expression level of CD28, CD107a, and ICOS in CD4+ and CD8+ T cells. (**D**) The expression level of CTLA4, LAG3, TIGIT, and TIM3 in CD4+ and CD8+ T cells. (**E**) The expression level of PD-L1 in activated DC, BDCA1- DC, BDCA1+ DC, and moDC cells. (**F**) The frequencies of live cells of B cells and moDC.

Furthermore, we aimed to analyze the baseline immunophenotype of PBMC of irMyocarditis patients in comparison to Ø tox ICI-patients before initiation of ICI-therapy. First, we determined the frequencies of activated T cells in peripheral blood. Here, CD103+ activated CD4+ T cells and CD103+ activated CD4+ Treg cells showed significant expansion in bl-irMyocarditis compared to bl-Ø tox ICI-patients,. whereas the abundance of CD103+ activated CD8+ T cell CD103+ activated CD8+ Treg cell were unaltered (Figures 5A,B). Regarding other phenotypes of T cells, we found no difference in frequencies of Tn, Tcm, Tem, and Teff neither in CD4+ nor in CD8+ T cells between bl-irMyocarditis patients and bl-Ø tox ICI-patients (Supplementary Figures S3A,B). To gain additional functional insights, we analyzed activation and exhaustion markers of the T cells. Interestingly, most activation markers showed significantly higher levels in bl-irMyocarditis, such as CD107a in CD8+ T cells, and ICOS in both CD4+ and CD8+ T cells, however CD28 was significantly higher in CD4+ T cells in bl-Ø tox ICI-patients compared to bl-irMyocarditis (Figure 5C). Expression of CD44, as well as CD27, showed no difference between bl-irMyocarditis and bl-Ø tox ICI-patients (Supplementary Figures S3C,D). Markedly, expression of the exhaustion marker LAG3 was highly elevated in both CD4+ and CD8+ T cells in bl-irMyocarditis. Significantly enhanced expression of CTLA4 in CD4+ T cells and TIGIT in CD8+ T cells, however a significant decrease of TIM-3 in CD4+, respectively, was detected in bl-irMyocarditis compared to bl-Ø tox ICI-patients (Figure 5D). In DC subsets, we observed an increased expression of PD-L1 on activated DC, BDCA1- cDC, BDCA1+ cDC, and moDC in bl-irMyocarditis patients (Figure 5E). Additionally, in our bl-irMyocarditis cohort, circulating B cells were significantly lower abundant than in bl-Ø tox ICI-patients. A significantly higher amount of moDC could be detected in the bl-irMyocarditis cohort compared to bl-Ø the tox ICI cohort (Figure 5F). To summarize, these data suggest that expression of exhaustion markers, particularly LAG3 on T cells and PD-L1 on DCs at baseline could be used as indicators to predict the occurrence of irMyocarditis.

## 4 Discussion

In recent years, the use of ICIs has been expanding due to their effectiveness in various tumor entities and even early tumor stages. Cardiotoxicity is a rare but often fatal side effect that is difficult to diagnose and hard to predict (23). Therefore, we aimed to investigate options for better patient monitoring including knowledge of pathogenesis and outcome under intensified management and to unravel potential immunophenotypes including suggestions for predictive signatures and transcriptomic pathways associated with development of cardiotoxicity.

In this multicenter study, we report on 51 patients with cardiac irAE induced by checkpoint inhibitor therapy. The analysis of laboratory results revealed elevated cardiac markers (Troponin, CK, CK-MB, NT-proBNP) in the majority of cardiac irAE patients. Subsequent examinations, such as ECG, Echo, LVEF, and cardiac MRI, commonly considered in the diagnostic work-up, were often nonspecific. But, as shown in Table 3, omission to perform further investigations is highly associated with an elevated mortality rate. Noteworthy changes were predominantly shown in cardiac biopsies. Since myocarditis is often a focal process in the myocardium especially in the early stages and due to the challenging procedure (24), we emphasize the need to be cautious of false-negative results. To further strengthen the validity of biopsy results and to minimize the risk of complications, those should be performed based on MRI [including late gadolinium enhancement (LGE)] findings.

In accordance with other reports with an onset of 30 days (25), irMyocarditis occurred early after ICI initiation, i.e., with a median of 36 days. Interestingly, in over 72% of patients another irAE occurred at the same time as the cardiac event. When cardiac irAEs are diagnosed, early initiation of high-dose steroids is of crucial importance (15). Patients with steroid-resistant irMyocarditis historically have the highest mortality rate of up to 43.7% (16). Treatment of steroid-refractory irAEs is largely based on expert opinions, which emphasizes the need for clinical studies. In our cohort, IVIGs were successfully applied as second-line immunosuppressants for cardiac irAE recalcitrant to steroids and led to high response rates as reported previously by Norwood et al. (26). While there are varying results on ICI rechallenge in cardiac irAE, with one case without recurrence of irAE, and another case, that resulted in mortality (3, 27), in 4 out of 5 patients from our cohort, re-initiation of ICIs after irAE resolution was feasible, while one patient died due to cardiac irAE upon ICI rechallenge. Nevertheless, better information including predictive markers in the challenging clinical setting of ICI rechallenge after cardiac irAE is still lacking.

Prior studies describe high fatality rates of over 39% for cardiac irAE (23). Our multi-center analysis, comparing different clinics’ approaches and the outcome of cardiac irAE, revealed that optimized management significantly reduces the mortality to 11.3% in our cohort. Therefore, we call for a standardized workflow in each center for the proactive, diagnostic, and therapeutic management of cardiac side effects, as prompt diagnosis and stringent monitoring significantly contribute to favorable outcomes. Shown in Supplementary Figure S4, we established an algorithm that could be applied across centers.

By comparing gene expression in cardiac biopsies of irMyocarditis patients to healthy heart muscle, we demonstrated an enrichment of inflammatory genes in irMyocarditis, especially highly significant for IL6R and STAT2. IL-6 has been described to increase during irAE and anti-IL-6 antibodies like tocilizumab have shown efficacy in treatment of irAE (28). Interestingly, this indicates potential parallels and might show validation on transcriptomic level for previous case reports with successful application of tocilizumab as anti-IL6R (29, 30) and of JAK-STAT-inhibitors (8, 16, 31) in severe cases with irMyocarditis. Additionally, we showed an upregulation of pathways related to cytokine-mediation, -response and -production in irMyocarditis patients. Since we also found an upregulation of the pathway of Epstein-Barr virus infection, we think this viral infection should be investigated as a potential risk factor for irMyocarditis development in future studies. Overall, our transcriptomic findings indicate as a distinct activation of the immune response in irMyocarditis with a need for further evaluation of especially anti-IL6R and JAK-inhibitor treatment.

Furthermore, we investigated immunophenotyping changes potentially related to irMyocarditis development. First of all, we demonstrated a higher activation of Treg cells and DCs in relation to the longitudinally observed development of irMyocarditis, explicitly shown by increased activated CD4+ Treg cells, enhanced level of PD-L1, and reduced levels of activated CD4+ T cells. Secondly, in comparison to patients without irAE undergoing ICI-therapy, patients with irMyocarditis showed a significant increase of activated Tem cells, with a higher expression of exhaustion markers (Supplementary Figure S5). There is a growing consensus that the response to ICI-therapy is achieved by modulating immunosuppressive cells such as Tregs (32). Recently, research demonstrated that Treg frequencies show a weak but statistically significant correlation with irAE severity (33). It also has been reported that patients with thymic epithelial tumor and non-small cell lung cancer show a strong increase of Tregs during anti-PD-1 therapy (34). Herein, patients with irMyocarditis show a higher frequency of Tregs compared to ICI-treated patients without toxicity. In the context of previous studies, our results of Tem cells might show parallels to the findings of Lozano et al. In this retrospective study, a strong correlation between the development of irAE and CD4+ Tem cells was identified (35). On the other hand, Tem (both CD4 and CD8) trended down in patients with irAE in a study investigated by Manfred, et al. (36). Additional work is needed to determine whether these different patterns represent mechanistic differences among different organ-specific irAE. Although the mechanisms of irMyocarditis are complex and incompletely understood, it is well established that irMyocarditis is mediated by the delicate regulation of the adaptive immune system. As their lineages of immune cells are interconnected, modulating the balance of their correlated T cell differentiation is a promising method for improving irMyocarditis.

The greatest challenges in the field of irMyocarditis surround monitoring, diagnosis and management, as well as the risks associated with re-challenge since decisions are based on expert opinion or retrospective data. Given the high mortality rate of irMyocarditis, an area of growing interest is biomarkers to predict irMyocarditis, like e.g., the composite biomarker score that includes the frequency of CD4 Tem cells in peripheral blood (35). Other biomarkers including cytokines, serum and other biological fluid proteins, genetic variations and gene profiles have been described as potential irMyocarditis predictors (22, 37–39). Our third finding regarding immunophenotyping indicated that T cell abundance could not serve as predictor for the development of cardiotoxicity. However, we observed LAG3 expression on T cells and PD-L1 expression on DC cells as potential predictive indicators for the occurrence of irMyocarditis comparing baseline immunophenotypes of irMyocarditis with patients without toxicity. Further studies will be needed to validate these proposed biomarkers.

## 5 Conclusion

The very low mortality rate of 11.3% for cardiac irAE in this cohort is remarkable and might be due to stringent monitoring since patients without troponin measurement were far more likely to die of the irAE. Our gene expression analyses imply an upregulation of IL6R and STAT2 and would thus suggest a more targeted therapy of the irAE with tocilizumab, or JAK-inhibitors, especially in steroid refractory irMyocarditis. Immunophenotyping of PBMC could enable early diagnosis of at risk groups that were characterized by enhanced Treg, Tem cells.

## Data Availability

The datasets presented in this study can be found in online repositories. The names of the repository/repositories and accession number(s) can be found in the article/**Supplementary Material**.
