# Supplementary material for: Stringent monitoring can decrease mortality of immune checkpoint inhibitor induced cardiotoxicity
Source: Front Cardiovasc Med. 2024 Jun 10;11:1408586. doi: 10.3389/fcvm.2024.1408586 (PMC11194425; doi:10.3389/fcvm.2024.1408586)
Supplement: Supplementary file 1 [file Presentation1.pdf]

Supplemental file 1 – Cardiac monitoring across different centers

Center	Cases (n)	Baseline investigations	Timepoints	Threshold for referral to cardiology	Further investigations					Deaths due to cardiac irAE (n), %
					**ECG**	**Echo**	**Coronary angiograpy**	**Cardiac MRI**	**Myocardial biopsy**	
**Cologne**	1	TnT, CK, CK-MB	**Prior** immunotherapy	**Increase** above the **baseline TnT** level before initiation of immunotherapy<br>**Significant TnT dynamics** in the short-term course<br>**New** cardio-pulmonary **symptoms**	Y	Y	Y (suspected myocardial damage)	Y (after exclusion of obstructive coronary artery disease)	(Y)	0/1 (0%)
		CK, (CK-MB)	Prior to **each** infusion							
		TnT;<br>ECG	Only with cardiac **symptoms**							
**Munich**	28	TnT, NT-proBNP, CK, CK-MB	**Prior** immunotherapy	**TnT** elevation (>0.014 ng/ml)<br>**New** cardiac **symptoms**	Y	Y	Y	Y	(Y)	2/28 (7.1%)
		TnT, NT-proBNP, CK, CK-MB	Prior to **each** infusion							
**Erlangen**	7	TnT, NT-proBNP, CK;<br>ECG, Echo	**Prior** immunotherapy	**TnT** elevation (>0.014 ng/ml)<br>**ECG** abnormalities<br>**New** cardiac **symptoms**	Y	Y	(Y)	(Y)	(Y)	2/7 (28.6%)
		TnT, NT-proBNP, CK;<br>ECG	Prior to **each** infusion							
**Essen**	1	TnI	**Prior** immunotherapy	**TnI** elevation (>0.045 ng/ml)	Y	Y	Y	Y	(Y)	0/1 (0%)
		TnI	Prior to **each** infusion for patients with combined immunotherapy and cardiac comorbidities							
**Freiburg**	1	None	**Prior** immunotherapy	**TnT** elevation (>0.021 ng/ml)<br>**ECG** abnormalities<br>**New** cardiac **symptoms**	Y	Y	Y (or CT-angiography)	Y	(Y)	0/1 (0%)
		TnT, NT-proBNP, CK;<br>ECG	Prior to **each** infusion **only** with **cardiac symptoms**							

Supplemental file 1 – Cardiac monitoring across different centers

**Hannover**	2	CK	**Prior** immunotherapy	**New cardiac symptoms** (and **TnT** elevation (>0.014 ng/ml))<br><br>**and/or ECG** abnormalities	Y	Y	Y	Y	Y	1/2 (50.0%)
		CK	Prior to **each** infusion							
**Luebeck**	1	Troponin,<br>24h-ECG,<br>cMRI or Echo	**Prior** immunotherapy	**Troponin** elevation (>1.5x upper limit of normal)<br><br>**New** cardiac **symptoms**	Y	Y	N	N	N	0/1 (0%)
		Troponin,<br>ECG	Prior to **each** infusion							
**Mainz**	3	None	Only with **cardiac symptoms**	Positive **Troponin** and increase of **NT-proBNP, CK, CK-MB**	Y	Y	Y	N	(Y)	1/3 (33.3%)
**Minden**	3	TnT, CK, NT-proBNP	**Prior** immunotherapy	Elevation of **TnT** (>0.014 ng/ml), **CK**, **NT-proBNP**<br><br>**New** cardiac **symptoms**	Y	Y	Y	Y	N	0/3 (0%)
		TnT, CK, NT-proBNP	Prior to **each** infusion							
**Nashville**	1	Troponin	**Weekly** for the first 3-4 weeks in patients treated with **combined** immunotherapy**, at each dose of nivolumab and relatlimab**<br><br>Only with **symptoms** for **PD-1 monotherapy**	Positive **Troponin**	Y	Y	N	Y	(Y)	0/1 (0%)
**Zuerich**	2	TnT, TnI, Myoglobin, CK, NT-proBNP; ECG, Echo	**Prior** immunotherapy	**TnT/TnI** elevation (>0.014 ng/ml)/(0.0156 ng/ml female and 0.0342 ng/ml male)<br><br>**ECG** abnormalities<br><br>**New** cardiac **symptoms**	Y	Y	Y	Y (or FDG-PET)	Y	0/1 (0%)
		TnT, TnI, Myoglobin, CK, NT-proBNP; ECG	Prior to **each** infusion							

Supplemental file 1 – Cardiac monitoring across different centers

Cardiac Monitoring across different centers. CK=Creatine Kinase, CK-MB=Creatine Kinase-MB, cMRI=cardiac Magnetresonanztomography, ECG=Electrocardiography, Echo=Echocardiography, FDG-PET=Fluorodeoxyglucose-Positron Emission Tomography, TnI=Troponin I, TnT=Troponin T.
