# Supplementary material for: Stringent monitoring can decrease mortality of immune checkpoint inhibitor induced cardiotoxicity
Source: Front Cardiovasc Med. 2024 Jun 10;11:1408586. doi: 10.3389/fcvm.2024.1408586 (PMC11194425; doi:10.3389/fcvm.2024.1408586)
Supplement: Supplementary file 2 [file Presentation2.pdf]

Supplemental file 2 – Basic characteristics of 11 irMyocarditis patients utilized in myocardial heart tissue analysis.

	Tumor entity	Age (Y)	Sex	Timing of endomyo-cardial biopsy	Number of days between first ICI administration and onset of irMyocarditis	Grade of irMyo-carditis	Basis for diagnosis of irMyocarditis	Other irAEs	IrAE outcome	Therapy of cardiac irAE
**1**	Breast cancer	56	Female	n/a	203	3	Cardiac symptoms; elevated troponin levels; pathological findings in cardiac MRI and endomyocardial biopsy	no	Ongoing	Corticosteroids
**2**	Melanoma	91	Female	Before steroid therapy	45	n/a	Pathological findings in endomyocardial biopsy	irHepatitis	Ongoing	Corticosteroids
**3**	Melanoma	69	Female	n/a	4	3	Cardiac symptoms; elevated troponin and NT-proBNP levels; pathological findings in cardiac MRI, coronary angiography and endomyocardial biopsy	Nephritis	Ongoing	Symptomatic therapy; Corticosteroids;
**4**	Melanoma	80	Male	n/a	12	3	Cardiac symptoms; elevated troponin and CK levels; pathological findings in ECG, echocardiography, cardiac MRI and endomyocardial biopsy	no	Resolved	Corticosteroids
**5**	Melanoma	30	Female	After steroid therapy	40	3	Cardiac symptoms; elevated troponin, NT-proBNP and CK levels; pathological findings in ECG, echocardiography, cardiac MRI and endomyocardial biopsy	no	Resolved	Corticosteroids
**6**	Neuro-endocrine bladder cancer	66	Male	n/a	35	4	Cardiac symptoms; elevated troponin, NT-proBNP and CK/CKMB levels; pathological findings in ECG, and endomyocardial biopsy	irHepatitis, irMyositis	Improved	Corticosteroids; Mycophenolate mofetil; Abatacept
**7**	Melanoma	56	Male	n/a	65	3	Elevated troponin, NT-proBNP and CK levels; pathological findings in ECG, cardiac MRI, coronary angiography and endomyocardial biopsy	no	Resolved	Symptomatic therapy; Corticosteroids
**8**	Melanoma	74	Female	n/a	19	3	Elevated troponin, NT-proBNP and CK levels; pathological findings in ECG and endomyocardial biopsy	irMyositis, irHepatitis, irColitis	Resolved	Corticosteroids; Intravenous immunoglobulins
**9**	Melanoma	73	Male	n/a	135	3	Elevated troponin, NT-proBNP and CK levels; pathological findings in ECG, echocardiography and endomyocardial biopsy	irHepatitis	Resolved	Symptomatic therapy; Corticosteroids
**10**	Melanoma	61	Female	n/a	27	3	Cardiac symptoms; elevated troponin, NT-proBNP and CK levels; pathological findings in ECG, cardiac MRI and endomyocardial biopsy	no	Resolved	Symptomatic therapy; Corticosteroids

Supplemental file 2 – Basic characteristics of 11 irMyocarditis patients utilized in myocardial heart tissue analysis.

**11**	Non-small-cell lung cancer (NSCLC)	58	Female	n/a	707	4	Cardiac symptoms; elevated troponin and CK levels; pathological findings in ECG, echocardiography and endomyocardial biopsy	no	Resolved	Symptomatic therapy; Corticosteroids

Basic characteristics of irMyocarditis patients. CK=Creatine Kinase, CK-MB=Creatine Kinase-MB, ECG=Electrocardiography, ICI=Immune checkpoint inhibitor, irAE=immune-related adverse event, MRI=Magnetic resonance imaging, N/A= not available, NSCLC=Non-small-cell lung cancer, NT-proBNP= N-terminal prohormone of brain natriuretic peptide, Y=years.
