# Supplementary material for: Stringent monitoring can decrease mortality of immune checkpoint inhibitor induced cardiotoxicity
Source: Front Cardiovasc Med. 2024 Jun 10;11:1408586. doi: 10.3389/fcvm.2024.1408586 (PMC11194425; doi:10.3389/fcvm.2024.1408586)
Supplement: Supplementary file 3 [file Presentation3.pdf]

Supplemental file 3 – Basic characteristics of 8 irMyocarditis patients used for longitudinal analysis of peripheral blood mononuclear cells (PBMCs) by flow cytometry

	Age (Y)	Sex	Timing of PBMC sampling (before or after steroid therapy, number of days after onset of irMyo-carditis)	Number of days between first treat-ment with ICI and onset of irMyo-carditis	Grade of irMyo-carditis	Basis for diagnosis of irMyocarditis	Other irAEs	Outcome	Treat-ment of irAEs
**1**	34	Male	Before	18	3	Cardiac symptoms; elevated troponin and CK levels; pathological findings in cardiac MRI	irSerositis	Resolved	Cortico-steroids
**2**	57	Male	Before<br><br>84 days after	34	4	Cardiac symptoms; elevated troponin, CK and NT-proBNP levels; pathological findings in ECG and cardiac MRI	irHypophysitis, irHepatitis	Resolved	Cortico-steroids
**3**	83	Fe-male	Before<br><br>75 days after	62	3	Cardiac symptoms; elevated troponin, CK and NT-prBNP levels; pathological findings in ECG	irThyroiditis, irPneumonitis	Resolved	Sympto-matic therapy
**4**	69	Fe-male	Before<br><br>18 days after	4	3	Cardiac symptoms; elevated troponin and NT-proBNP levels;<br>pathological findings in echocardiography, cardiac MRI and endomyocardial biopsy	irNephritis	Ongoing	Sympto-matic therapy; Cortico-steroids
**5**	82	Male	Before	140	3	Elevated troponin level	irColitis irHepa-titis	Resolved	Cortico-steroids
**6**	77	Male	Before	59	3	Elevated troponin, CK, CKMB and NT-proBNP levels; pathological findings in ECG, echocardiography and cardiac MRI	No	Ongoing	Cortico-steroids
**7**	28	Male	Before<br><br>30 days after	203	3	Cardiac symptoms; elevated troponin level; pathological findings in ECG and cardiac MRI	No	Resolved	Sympto-matic therapy
**8**	56	Fe-male	Before	30	3	Cardiac symptoms; elevated troponin and NT-proBNP levels; pathological findings in ECG, cardiac MRI and endomyocardial biopsy	No	Ongoing	Cortico-steroids

Basic characteristics of irMyocarditis patients. CK=Creatine Kinase, CK-MB=Creatine Kinase-MB, ECG=Electrocardiography, ICI=Immune checkpoint inhibitor, irAE=immune-related adverse event, MRI=Magnetic resonance imaging, N/A= not available, NT-proBNP=N-terminal prohormone of brain natriuretic peptide Y=years.
