# Supplementary figures and images for: Stringent monitoring can decrease mortality of immune checkpoint inhibitor induced cardiotoxicity
Source: Front Cardiovasc Med. 2024 Jun 10;11:1408586. doi: 10.3389/fcvm.2024.1408586 (PMC11194425; doi:10.3389/fcvm.2024.1408586)

# Suppl. Fig.1

(A)

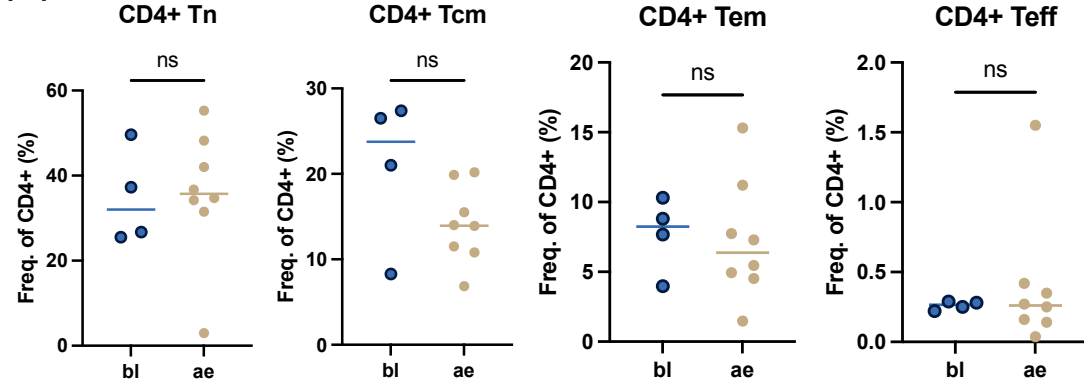

(B)

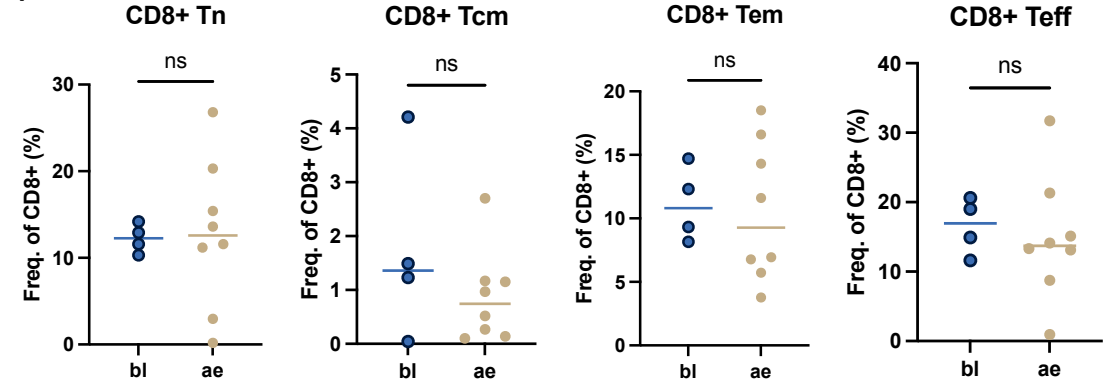

(C)

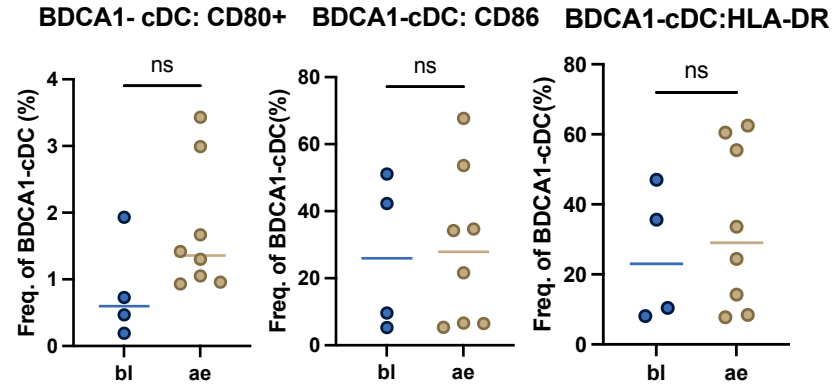

(D)

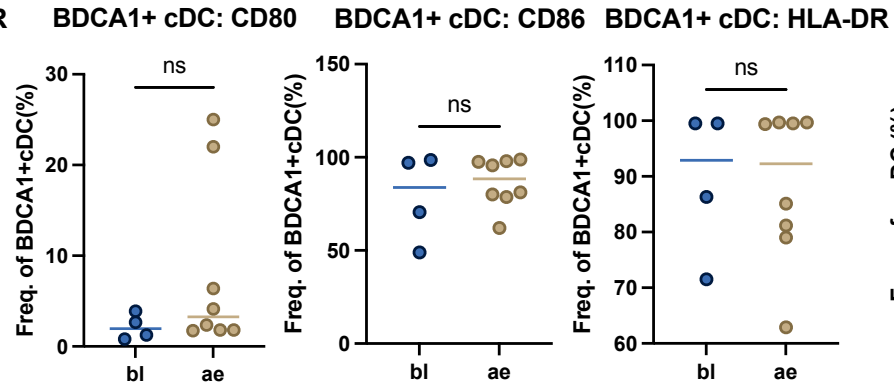

(E)

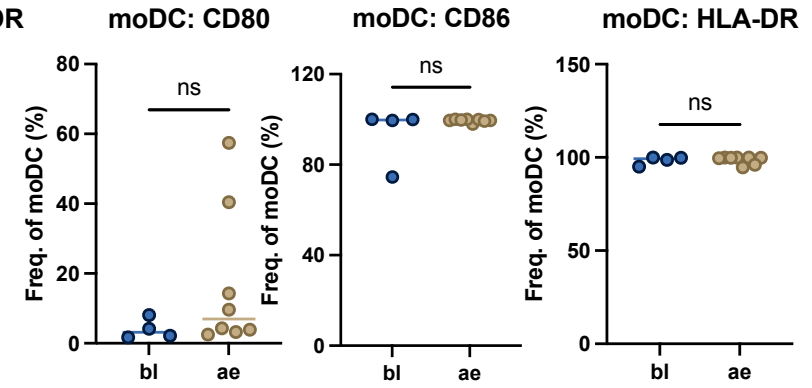

# Suppl. Fig.2

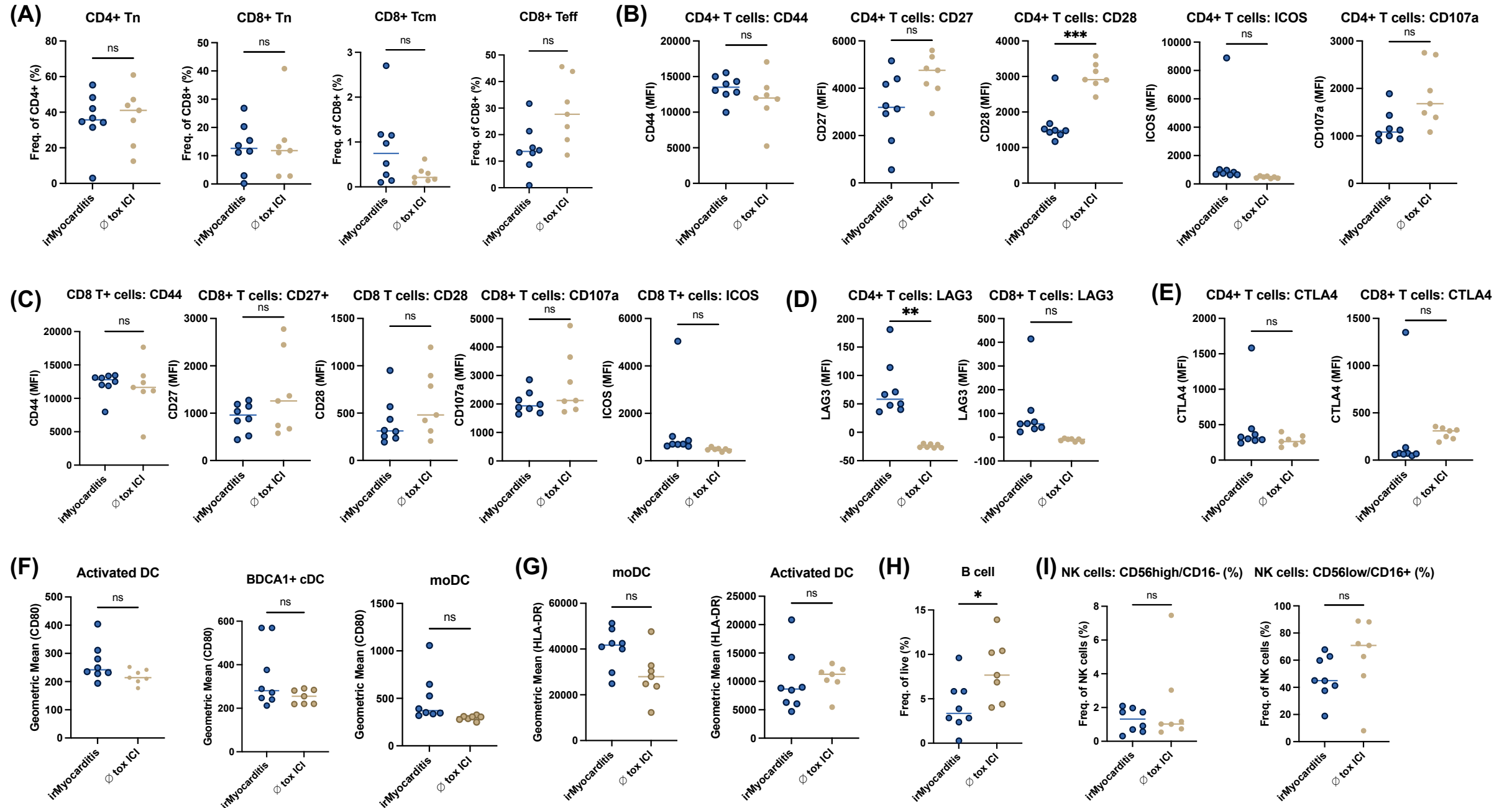

# Suppl. Fig.3

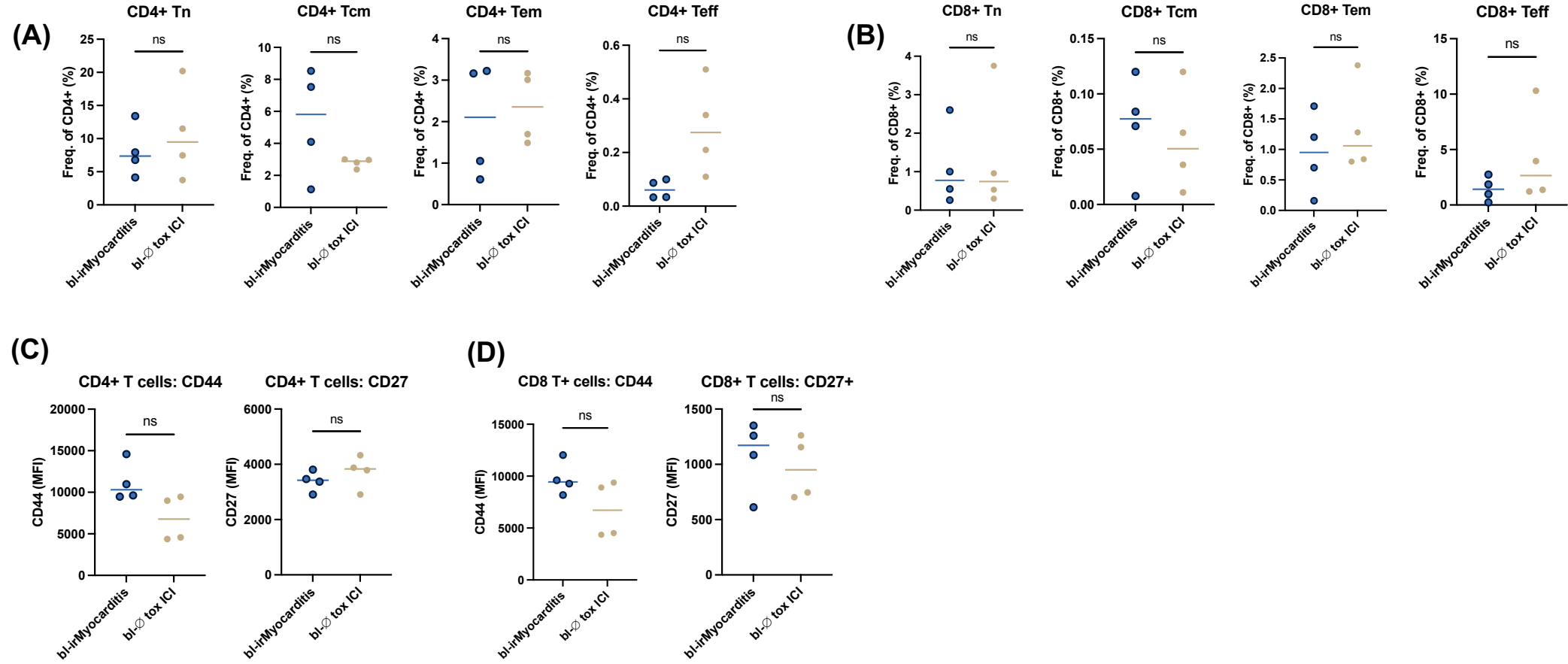

Suppl. Fig.4

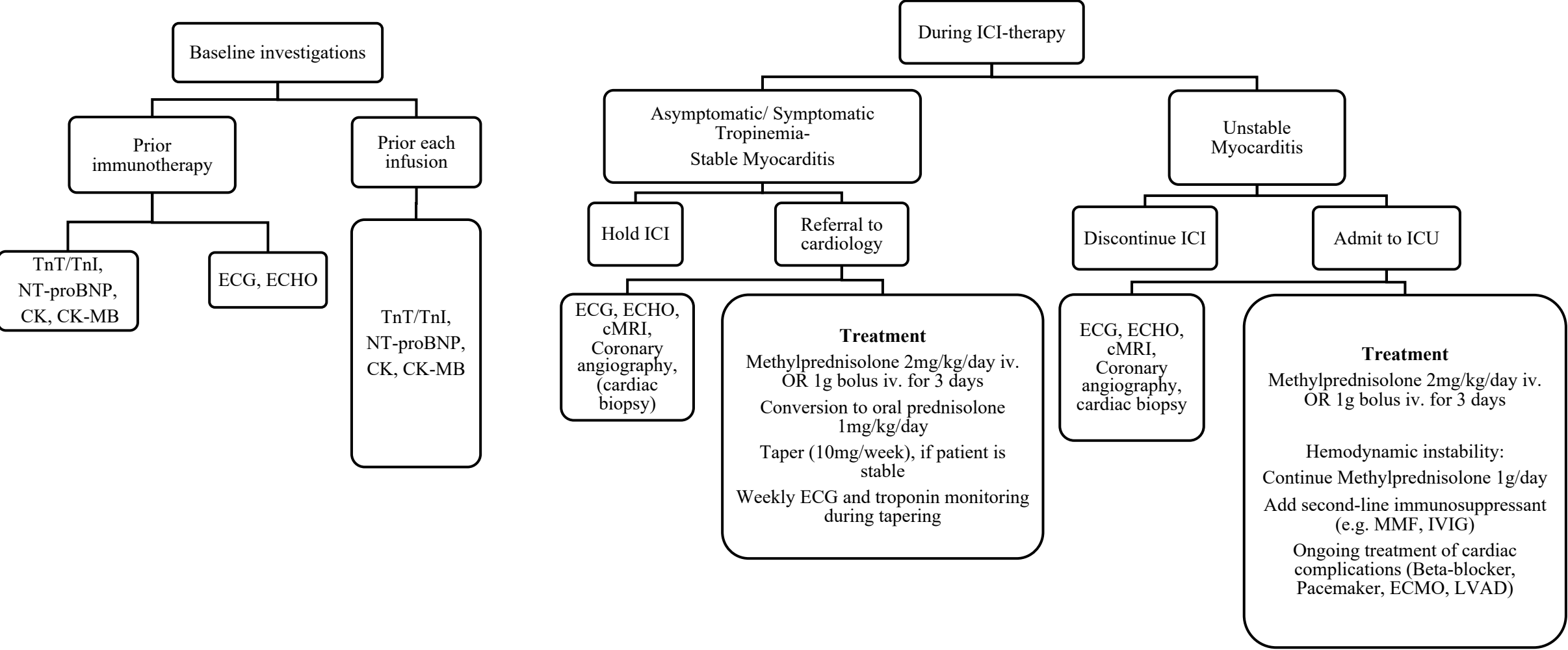

Suppl. Fig.5

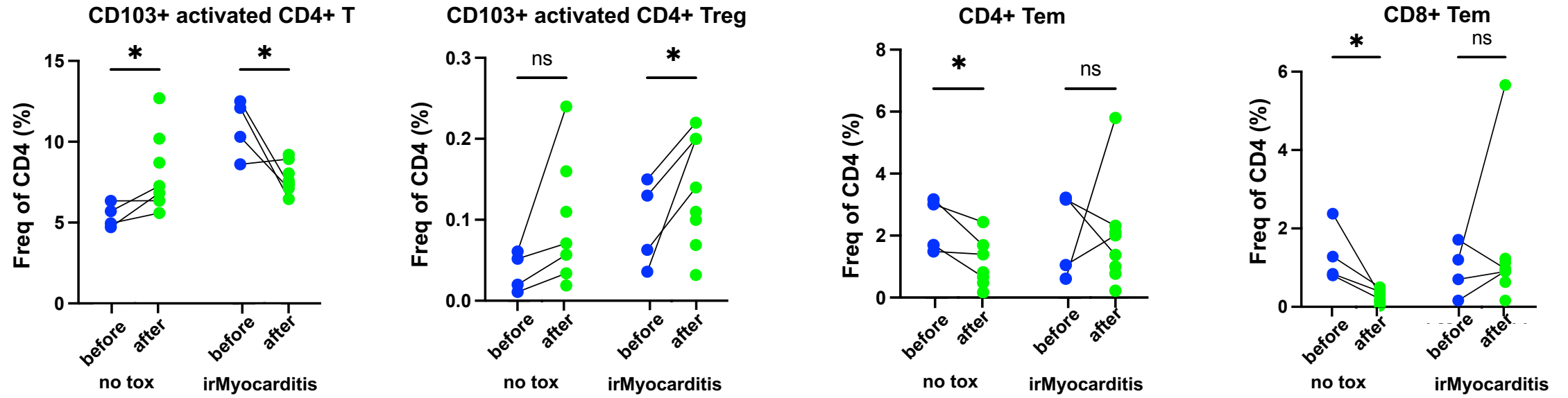

Supplement: Supplementary file 4 [file Image1.pdf]
